# Treatment of Wounds of the Eye

**Published:** 1854-10

**Authors:** 


*Treatment of Wounds of the Eye.*—Modern ophthalmic surgery has very considerably deviated from what used to be the usual practice in case of wounds, either incised or punctured, of the eye. In a large majority of cases it was formerly considered necessary to employ pretty active antiphlogistic measures in order to obviate the much-dreaded effects of inflammation. As a consequence of this habit, frequent examinations of the organ, in order to watch what was going on, became necessary. It is now known that, in all such cases, there must of necessity follow considerable inflammation. A degree of congestion and the effusion about the injured parts of much soft lymph are, therefore, looked upon as matters of course, and as stages of the healing process not to be interfered with. The great principle of the treatment which we see pursued at the Royal London Ophthalmic Hospital is to keep the injured organ completely at rest. Almost every one is experimentally familiar with the degree of comfort, in cases of slight scratches of the eye, or when dust has got into it, which is afforded by gently supporting the closed lids with the hand. So long as the lids are kept close and still, the pain is absent, but the moment any movement is attempted, a sharp stab of pain is caused. By extending the knowledge thus gained to the more severe injuries, we arrive at the principle alluded to. The following is the detail of the measures usually adopted at the Institution named.—On the patient's first application, the eye is carefully inspected; if any foreign matters are discovered, they are removed; if any portion of the front of the cornea have been partially detached, or if any considerable portion of iris have been protruded so as to be irreducible, the piece is snipped away by means of small scissors. It is rare that anything can be done in improving the position of the edges of the wound, or in returning protruded structures; and the next step is, therefore, to close the lids, and, having carefully padded them with layers of cotton-wool, the whole is confined by strips of adhesive plaster. A mild dose of aperient medicine is generally given, and the patient directed to live carefully on a rather less stimulating diet than ordinary. Unless urgent symptoms make it necessary earlier, which is very rare, the eye is not again inspected for several days, or perhaps a week, when the dressings are cautiously removed and replaced. It is very infrequent indeed for any depletory measures, either topical or general, to be deemed necessary. The symptoms which would excite alarm, and be held to indicate the necessity for further examination, are severe tensive or throbbing pain in the globe, or general fever; and, in the absence of both these, it is judged certain that interference cannot be needed, and that frequent inspections of the organ could but be productive of injury.

We may remark, that the plan of padding the lids and keeping the eye closed is by far the best which can be pursued, after the removal of dirt, etc., from the eye, or after slight injuries to it from blows or scratches. Under such circumstances it is rarely required more than a day or two.—*Med. Times.*

